# The heterogeneous impact of public security cameras on safety perceptions in cities: Evidence from China

**DOI:** 10.1093/pnasnexus/pgaf331

**Published:** 2025-10-16

**Authors:** Pinghan Liang, Yadi Liu, Yuchen Guo, Fanqi Zeng

**Affiliations:** Center for Chinese Public Administration Research, Sun Yat-sen University, Guangzhou 510275, China; School of Government, Sun Yat-sen University, Guangzhou 510275, China; School of Government, Sun Yat-sen University, Guangzhou 510275, China; School of Government, Sun Yat-sen University, Guangzhou 510275, China; Department of Sociology, University of Oxford, Oxford OX1 1JD, United Kingdom

**Keywords:** public safety, safety perception, societal sustainability, urban development

## Abstract

Digital public security systems in cities, such as surveillance cameras, can record illegal behavior and deter crime, thereby enhancing residents’ perceptions of safety. However, the impact of public surveillance cameras on residents’ safety perceptions in urban environments remains insufficiently understood, especially at the national level. Drawing on a novel dataset from Chinese government procurement records on surveillance cameras (2013–2017) and a representative national household survey, we find that a 10% increase in per capita camera expenditure results in a 0.2-unit increase in residents’ perceived safety. Notably, this effect is observed only among local residents. For migrants, surveillance cameras improve perceived safety only when complemented by social ties, with neighborhood ties being more effective than distant ones in shaping these perceptions. Furthermore, the effectiveness of surveillance cameras on migrants’ perceived safety is moderated by weather conditions and the inclusiveness of the urban environment. Our findings highlight that while public security technology can improve residents’ safety perceptions, human-centered development remains essential for sustainable urban governance.

Significance Statement Urban residents’ perceptions of safety are influenced not only by technology but also by local social connections. This research examines how public surveillance cameras, widely used in cities to deter crime, affect residents’ safety perceptions on a national scale. By analyzing national data on camera investments and a large household survey from China, the study reveals that public surveillance cameras can enhance safety perceptions among local residents in cities. However, for migrants, who often lack strong community ties, these cameras improve safety perceptions only when paired with robust local social networks. These findings suggest that technological security measures should be integrated with efforts to strengthen social bonds to create more inclusive and sustainable urban environments.

Crime and violence in cities pose a direct threat to individual safety, causing fear among residents (1–4). In response, public surveillance cameras—automated, nonpersonalized detection technologies—have been widely deployed by various stakeholders in settings such as commercial centers and parking lots (5). These systems preserve evidence of crime and enhance the detection of criminal activities, thereby deterring potential offenders (6) and bolstering public perceptions of safety (7). Surveillance cameras have been adopted as a central element of crime prevention strategies in numerous countries, including the United States, Colombia, and China (8). Government-operated surveillance often entails the collection and processing of personal data, raising concerns about privacy. Such concerns may induce unintended consequences, including a chilling effect (9), whereby individuals alter their behavior in response to perceived constant monitoring—ultimately weakening the intended gains in public safety and order (10).

Public acceptance of surveillance technologies also varies across institutional contexts. In China, survey-based evidence suggests that residents tend to express relatively high levels of support for surveillance systems (11, 12). This may reflect a combination of political trust associated with a “guardian” governance model and a widespread belief in the effectiveness of surveillance for crime prevention. Against this backdrop, we here conceptualize the “sense of safety” as a subjective perception centered on fear of crime but shaped by broader institutional, economic, and cultural influences, such as governance practices, living costs, and social inclusion.

Beyond crime deterrence, surveillance cameras have become central instruments of urban governance, helping to maintain public order and safeguard residents’ sense of safety. Many are deliberately installed in visible locations and accompanied by explicit warnings, such as “You are under surveillance” or “CCTV in operation,” to reinforce their deterrent presence. These systems facilitate the detection of traffic violations, discourage unauthorized gatherings, and help prevent street-level disorder. Nevertheless, their effectiveness is limited by adaptive offender behavior, such as the displacement of criminal activity to unmonitored areas (13), which may undermine broader goals of public order.

Moreover, the sense of safety often stems from the care and vigilance of individuals dedicated to the collective protection of their communities (14, 15), which can also be influenced by the presence of surveillance cameras. As a result, in some cases, these cameras may even create a false sense of security among residents (16). Overall, the existing literature on the impact of surveillance cameras on urban residents’ perceived safety remains inconclusive, with evidence reflecting mixed outcomes (17–19).

In addition to the advancements in the digital security technologies, social factors such as trust and cohesion, formed through individuals’ social ties, play a crucial role in enhancing safety perceptions within urban neighborhoods (20, 21). Close social ties enable individuals to collectively enforce and adhere to shared norms of public behavior (22, 23). This collective efficacy not only reduces crime but also strengthens individuals’ perceptions of safety (24–26). Social ties, in particular, provide timely assistance in dangerous situations (17, 18), and offer an advantage over surveillance cameras by mitigating privacy concerns (7). While social ties can significantly foster a sense of safety, maintaining distant social networks often demands greater time and emotional investment (14).

Besides, different groups possess varying degrees of social ties, particularly marginalized groups, which are often considered vulnerable populations (27). Among these marginalized groups, urban migrants frequently encounter significant barriers to equitable access to the benefits of urban development, including employment, housing, and healthcare (28, 29). Yet, it is not clear whether surveillance cameras can help them overcome these disadvantages and promote their perception of safety (30, 31). Previous studies, largely based in Western contexts, has paid little attention to how digital surveillance intersects with migrants’ structural vulnerabilities and the geographic proximity of social ties in shaping perceived safety. Whether such intersections can enhance migrants’ sense of safety in the Chinese context remains an open question.

Meanwhile, much of the existing research on public safety perceptions under surveillance cameras in cities (7, 27, 32–34) relies on qualitative surveys conducted in limited locations or small-scale case studies. Large-scale or national-level investigations into urban public safety perceptions are rare, primarily due to data constraints and the difficulty of identifying cities with suitable surveillance camera infrastructure. The effects of surveillance cameras on urban residents’ perceived safety, specifically how migrants perceive safety in cities, have not yet been thoroughly examined using large-scale observational data.

China, as a Global South country with a population exceeding 1.4 billion in 2023 (35), 65% of whom reside in urban areas (36), has experienced rapid urbanization since 2000 (37). The country has also established the world’s largest surveillance camera network, with ∼200 million cameras installed in public spaces by 2018 (38), providing a unique context for examining the large-scale effects of surveillance cameras on public safety perceptions (39). Notably, China’s exceptionally low homicide rate of just 0.46 per 100,000 population (40) contrasts sharply with the global average of 5.6 per 100,000 (41), positioning the country among those with the lowest crime rates around the world recently.

In this study, we utilize two waves of the China Labor Force Dynamic Survey (CLDS) (42), conducted in 2016 and 2018 (N=11,411), a longitudinal dataset focused on China’s labor force, in conjunction with a dataset of 22,572 surveillance camera procurement contracts signed by public security departments across China, which we collected from local government websites, covering 290 cities between 2013 and 2017. This combined dataset offers a unique opportunity to examine the impact of surveillance cameras on the safety perceptions of urban residents in China.

We apply econometric models to these large datasets and derive three key findings. First, surveillance cameras significantly reduce urban residents’ fear of crime and enhance their overall safety perception in Chinese cities, with a 10% increase in per capita surveillance camera value leading to a 0.2-unit increase in perceived safety. Instrumental variable estimation confirms the robustness of this finding by addressing endogeneity concerns. However, this effect is confined to local inhabitants and does not extend to migrants. Second, only migrants with strong local social ties experience an improvement in safety perception from the presence of surveillance cameras. Third, extreme weather conditions and inadequate nighttime lighting reduce the effectiveness of cameras for migrants, while urban inclusiveness enhances their impact.

Our findings make the following contributions. First, we provide the nationwide evidence on the effectiveness of surveillance cameras on public safety perceptions, extending the understanding of the benefits and costs associated with the large-scale deployment of digital security technology in cities. Second, we illustrate the unequal effects of surveillance cameras on perceived safety, highlighting the potential distributional consequences of digital security technology across different demographic groups. This has important policy implications for identifying and helping vulnerable populations to enhance their safety perceptions. Third, we demonstrate that surveillance cameras only partially substitute for community and interpersonal interactions in fostering safety perceptions. This underscores the indispensable role of human efforts and community policing in maintaining social order in cities.

## Results

### Populated and developed cities have more surveillance camera procurement in China

**Fig. 1.**
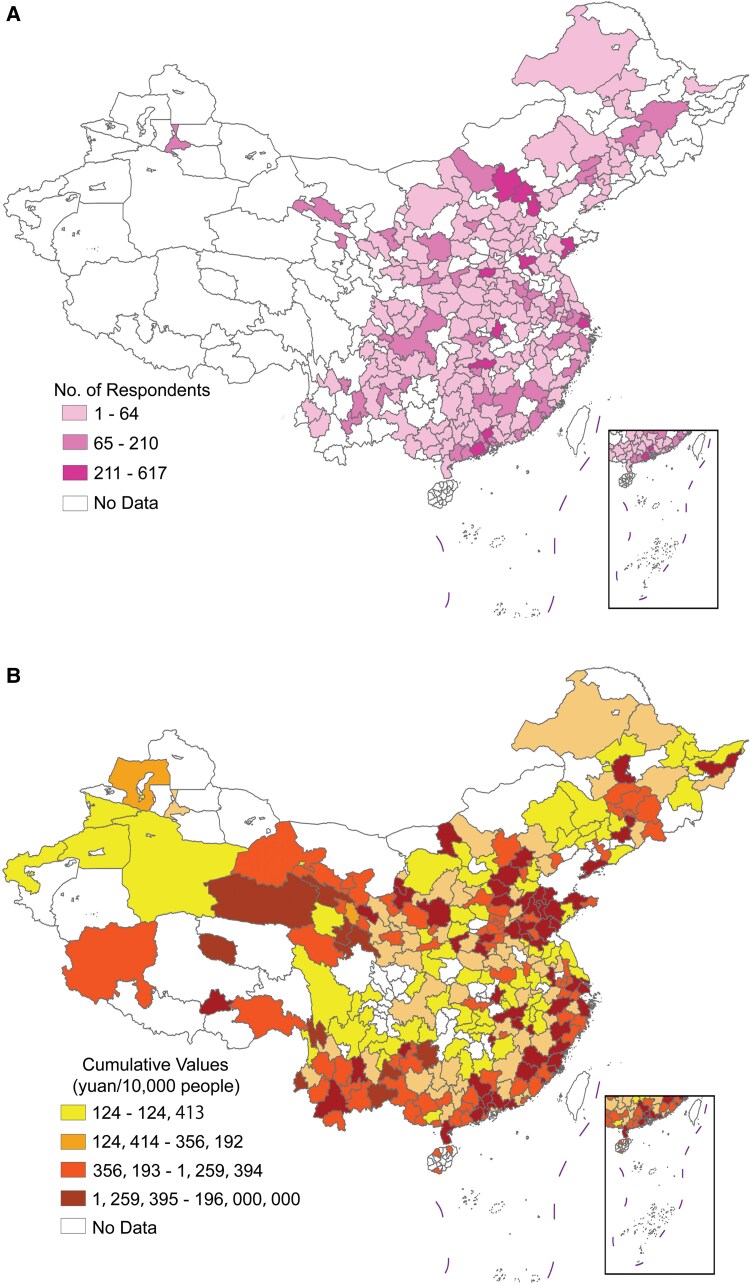
Geographic distributions of CLDS-sampled cities and cities with surveillance camera procurement. A) Colored areas represent cities (N=206) sampled by the CLDS, with the number of survey respondents indicated in the legend. B) Colored areas represent cities (N=290) that had surveillance camera procurement between 2013 and 2017, with darker colors indicating higher per capita cumulative procurement values.

We first map the geographic distribution of CLDS-surveyed cities (N=206) alongside the sampled cities (N=290) involved in surveillance camera procurement in China. As shown in Fig. 1A, the surveyed cities are predominantly located in the eastern and central regions, with a notable concentration in areas with high urban populations and strong economic development, such as the Yangtze River Delta (a major metropolitan area centered around Shanghai on the east coast) and the Pearl River Delta (a major metropolitan area centered around Guangzhou on the south coast). Additionally, there is substantial coverage in northern China, including parts of Northeast China. The distribution of urban respondents is relatively even, with no significant clustering evident.

Figure 1B displays the geographic density of aggregated surveillance camera procurement across China between 2013 and 2017, which is similarly concentrated in economically developed and densely populated cities (N=290). For example, cities in the Yangtze River Delta and Pearl River Delta regions, as well as most provincial capitals, exhibit procurement patterns closely aligned with the distribution of CLDS-surveyed cities. In contrast, certain western regions with lower population densities, such as Tibet and Xinjiang, show relatively lower aggregate values of surveillance camera procurement. Additionally, we present the yearly total procurement value of urban surveillance cameras in China from 2013 to 2017 (see Fig. S2).

### Local population exhibits stronger social ties and higher safety perception compared to migrants

**Fig. 2.**
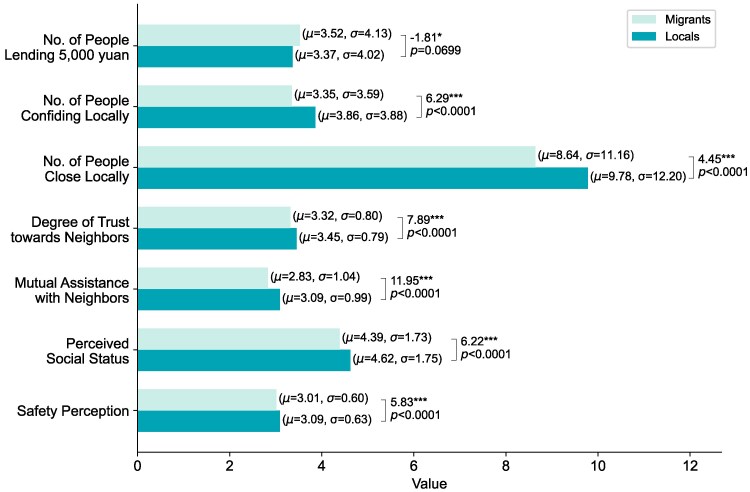
Histogram of local and migrant population samples. The local population sample consists of 8,426 individuals, while the migrant population sample includes 2,949 individuals. The length of each bar represents the mean value of the corresponding variable, with the mean (*μ*) and SD (*σ*) provided in parentheses next to the bar. A two-sided t-test is conducted to compare local and migrant groups for each variable, with the t-statistic and *P*-value displayed at the right end of the bars. ***P<0.01, **P<0.05, *P<0.1.

We classify social relationships into geographically dispersed local social ties and concentrated community social ties (26), further distinguishing between instrumental ties, such as financial support, and emotional ties, such as confiding personal thoughts to others (43). Figure 2 illustrates the differences between local and migrant populations in cities in terms of the strength of social ties and perceived safety. Overall, local residents tend to have stronger social ties and report a higher sense of safety compared to migrants in urban areas of China.

In the context of social vulnerability, the fear of criminal behavior is closely tied to the heightened sense of insecurity experienced by migrants, often resulting from institutional marginalization (44, 45). For migrants in cities, social ties play a critical role in providing daily practical support (46). However, the migration experience can sever many social connections and support systems, creating a “relationship deficit” (47), which often leads migrants to perceive their own social status as lower (48). Interestingly, in terms of instrumental social ties, such as borrowing money, migrants exhibit a higher average level than local residents (Fig. 2). Migrants, who often face greater financial pressures in their new environments while seeking employment opportunities, are more likely to form social connections that offer economic assistance.

### Surveillance cameras enhance locals’ perceived safety but have limited impact on migrants

Table 1 presents the impact of surveillance cameras on residents’ safety perceptions, estimated using ordinary least squares (OLS) regressions. Model (2), which includes all controls and fixed effects, shows that a 10% increase in surveillance cameras is significantly associated with a 0.2-unit increase in perceived safety, equivalent to one-third of a SD. Models (3)–(6) indicate that surveillance cameras significantly enhance the perceived safety of local residents, but no significant effects are observed among migrants. The interaction term in model (7) further confirms this pattern. These findings suggest that surveillance cameras, as a form of formal social control, have a limited impact on migrants’ perceptions of safety. Compared to local residents, migrants’ perceptions of safety and their views on surveillance cameras are more nuanced. Due to their limited familiarity with the host city’s surveillance infrastructure and the social integration challenges they face, migrants have a diminished capacity to derive a sense of security from such technical safety measures.

**Table 1.** OLS results of baseline regressions.

Variable	Safety perception						
	All sample	All sample	Migrants	Local	Migrants	Local	All sample
	(1)	(2)	(3)	(4)	(5)	(6)	(7)
ln(per capita procurement value)	0.012**	0.020***	− 0.002	0.011**	0.005	0.020***	0.021***
	(0.005)	(0.005)	(0.009)	(0.005)	(0.010)	(0.006)	(0.005)
Migrants							0.080
							(0.074)
Cameras * Migrants							−0.010*
							(0.006)
Individual characteristics		✓			✓	✓	✓
Family characteristics		✓			✓	✓	✓
Community characteristics		✓			✓	✓	✓
City characteristics		✓			✓	✓	✓
Year * Province		✓			✓	✓	✓
Empirical *P*-value	-	-	0.000***		0.005**		–
*N*	10,005	9,628	2,667	7,338	2,540	7,088	9,628
Adjusted $R^{2}$	0.041	0.07	0.03	0.048	0.06	0.077	0.071

Note: Table 1 reports the coefficient estimates from the OLS models. A ✓indicates that control variables and spatial fixed effects are included in the model. Models (1) and (2) present the effects of surveillance cameras on the perceived safety of all individuals. However, the impact of surveillance cameras may vary between local residents and migrants. Therefore, models (3) through (6) provide subgroup regression results. To better capture group differences and enhance the robustness of the baseline results, model (7) incorporates interaction terms to assess the variation between groups. Standard errors (in parentheses) are clustered at the individual level. ***P<0.01, **P<0.05, *P<0.1.

As noted earlier, the effectiveness of surveillance cameras is, to some extent, conditioned by the relatively high level of trust that Chinese citizens place in the government (12). Our supplementary analysis further reveals the critical role of institutional trust in shaping residents’ perceptions of safety (see details in Table S1). Specifically, surveillance cameras are significantly effective in enhancing perceived safety in cities with higher levels of trust in local authorities, while their impact is negligible in low-trust settings. These findings suggest that surveillance technologies, as instruments of urban governance, derive their protective value not solely from their technical capabilities but from being embedded within broader contexts of legitimacy and public confidence in state institutions.

To address the endogeneity challenge, we use previous collective protest events as an instrumental variable (49, 50) (see Table S2). Additionally, we revised the measurement criteria for migrants for robustness testing. Given that recent migration experiences may lead to more complex safety perceptions, encompassing factors like adaptation to new environments and social integration, which could confound the evaluation of the impact of surveillance cameras, we excluded individuals who had migrated within the current year from the analysis. To further mitigate potential confounding effects, we controlled for cumulative crime levels in cities prior to 2015, as these could influence both the installation of surveillance cameras and residents’ perceptions of safety. Finally, we aggregated individual safety perceptions at the city level to calculate the average overall perception of safety. The results of these analyses are presented in Tables S3.1 and S3.2.

### Surveillance cameras reduce locals’ fear of crime but have limited impact on migrants

We examine the relationship between surveillance cameras and fear of crime among local residents and migrants to further clarify the mechanisms underlying perceived safety. Specifically, we distinguish among three dimensions of crime-related fear: walking alone at night, residential burglary, and being targeted for displaying wealth. All three are measured using ordered scales ranging from 1 (No fear) to 6 (Extreme fear), reflecting increasing levels of perceived threat. We begin by presenting descriptive statistics of the three types of crime-related fear among local residents and migrants (see Table S4). The results show that, on average, migrants report higher levels of fear compared to locals. However, this difference is statistically significant only in the case of residential burglary.

**Fig. 3.**
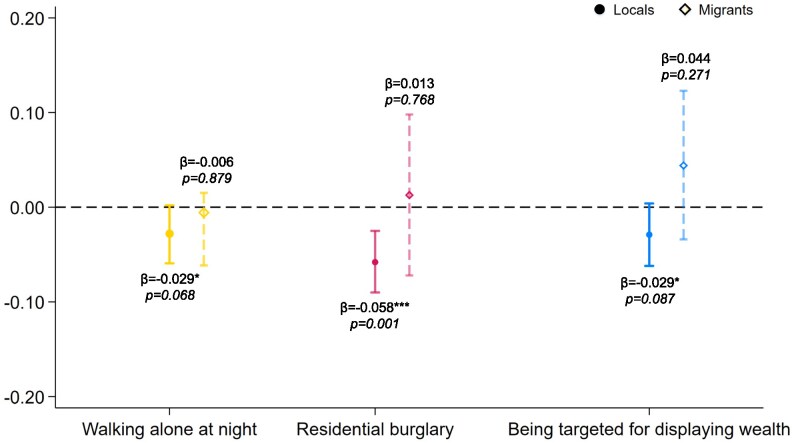
Effects of surveillance cameras on fear of crime. The figure shows the estimated effects (*β*) of surveillance camera density on three dimensions of fear of crime. Solid circles represent local residents (N=3,136), while hollow diamonds represent migrants (N=993). Vertical lines indicate 90% CI. A negative coefficient suggests that increased surveillance coverage is associated with reduced fear. ***P<0.01, **P<0.05, *P<0.1.

Figure 3 reveals a clear divergence between locals and migrants. Among local residents, higher surveillance camera density is significantly associated with reduced fear across all three crime types. The strongest effect is observed for residential burglary (β=−0.058, P=0.001), suggesting that a 10% increase in per capita surveillance camera expenditure corresponds to a 0.058-unit decrease in burglary-related fear. These results indicate that locals interpret surveillance infrastructure as both a credible deterrent to property crime and a visible indicator of institutional control, particularly in relation to family property security. In contrast, we find no statistically significant association between surveillance camera density and crime-related fear among migrants. This asymmetry highlights the critical role of social and institutional embeddedness in shaping how individuals interpret public security measures. These findings underscore that migrants’ structural vulnerability rooted in social marginalization amplifies fear of crime and weakens the perceived protective value of surveillance infrastructure.

### Social ties and surveillance cameras jointly enhance migrants’ safety perception

Table 2 examines the relationship between surveillance cameras and migrants’ social ties in shaping their safety perceptions. The positive and significant interaction terms in models (1) and (2) indicate that surveillance cameras, when complemented by strong local emotional ties, enhance migrants’ perceived safety. These results suggest that for migrants with well-developed local emotional and social ties, the presence of surveillance cameras has a greater positive effect on their safety perception. Similarly, the interaction between neighborhood social ties and surveillance cameras further supports this complementary effect, with models (4) and (5) showing that the impact of neighborhood social ties is even more pronounced than that of local social ties. This finding underscores the value of social relationships in reducing perceived insecurity.

**Table 2.** OLS results of the impact of social ties.

Variable	Safety perception				
	Migrants	Migrants	Migrants	Migrants	All sample
	(1)	(2)	(3)	(4)	(5)
ln (per capita procurement value)	0.006	0.006	0.003	0.011	0.006
	(0.010)	(0.010)	(0.011)	(0.010)	(0.010)
No. of local close contacts	− 0.000				
	(0.001)				
Procurement value * No. of local close contacts	0.001**				
	(0.000)				
No. of local confidants		− 0.003			
		(0.003)			
Procurement value * No. of local confidants		0.002*			
		(0.001)			
No. of local persons who can lend money			0.003		
			(0.003)		
Procurement value * No. of local persons who can lend money			0.001		
			(0.001)		
Mutual assistance with neighborhood				0.077***	
				(0.013)	
Procurement value * Mutual assistance with neighborhood				0.010*	
				(0.005)	
Trust towards neighborhood					0.138***
					(0.017)
Procurement value * Trust towards neighborhood					0.014**
					(0.007)
Individual characteristics	✓	✓	✓	✓	✓
Family characteristics	✓	✓	✓	✓	✓
Community characteristics	✓	✓	✓	✓	✓
City characteristics	✓	✓	✓	✓	✓
Year * Province	✓	✓	✓	✓	✓
*N*	2,540	2,540	2,540	2,540	2,540
Adjusted $R^{2}$	0.060	0.060	0.060	0.077	0.095

Note: Table 2 reports the impact of social ties on the safety perceptions of 2,540 migrant respondents. Models (1) to (3) examine the effect of local social ties on migrants’ safety perception. Specifically, model (1) presents the interaction between surveillance cameras and the number of close local connections migrants have. Model (2) shows the interaction between surveillance cameras and the number of locals in whom migrants confide. Model (3) examines the interaction between surveillance cameras and the number of individuals from whom migrants can borrow money. Models (4) and (5) focus on the influence of neighborhood social ties on migrant safety perception. Model (4) reports the interaction between surveillance cameras and the level of neighborhood mutual assistance, while model (5) shows the interaction between surveillance cameras and neighborhood trust. Coefficient estimates are obtained using OLS regressions, with standard errors (in parentheses) clustered at the individual level. ***P<0.01, **P<0.05, *P<0.1.

More importantly, our study highlights the significant role of shifting social ties from the broader local level to the more intimate neighborhood level in influencing migrants’ safety perceptions. The accessibility of support services is crucial when facing criminal threats. The presence of surveillance cameras, combined with strong social bonds, provides a dual layer of perceived safety—both technological and emotional. Together, these factors demonstrate how technological tools and social ties can work in tandem to improve community safety, thereby increasing migrants’ sense of security in their environment.

However, the interaction term for instrumental local social ties is statistically insignificant (model (3)). This result is not surprising. Emotional social ties provide long-term stability and a comprehensive safety net that extends beyond mere economic or practical support. These ties are grounded in trust and emotional support, fostering deep personal connections that enhance a sense of belonging, which, in turn, alleviates anxiety and insecurity. In contrast, instrumental relationships, which are transactional by nature, are more subject to fluctuations in economic needs and utility, making them less effective in contributing to a sustained sense of security.

### Urban climate and ambient lighting heterogeneity

Safety perception, as a subjective experience, is influenced by both individual characteristics and environmental conditions (51). Extreme weather, in particular, can affect crime rates through both economic and psychological mechanisms, thereby influencing how effective surveillance infrastructure is in enhancing public safety perceptions. For instance, droughts can reduce agricultural income and intensify economic hardship, lowering the opportunity cost of engaging in crime (52). In contrast, rainfall tends to reduce street crime by discouraging outdoor activity and impairing visibility (53, 54). Temperature fluctuations also affect emotional states; high temperatures, for example, are linked to increased irritability, which may elevate the incidence of violent crimes. Recent evidence further indicates that the relationship between temperature and crime is nonlinear: while moderate warmth may encourage social interaction and increase opportunities for crime, extreme heat tends to suppress outdoor activity and reduce criminal behavior (55).

**Fig. 4.**
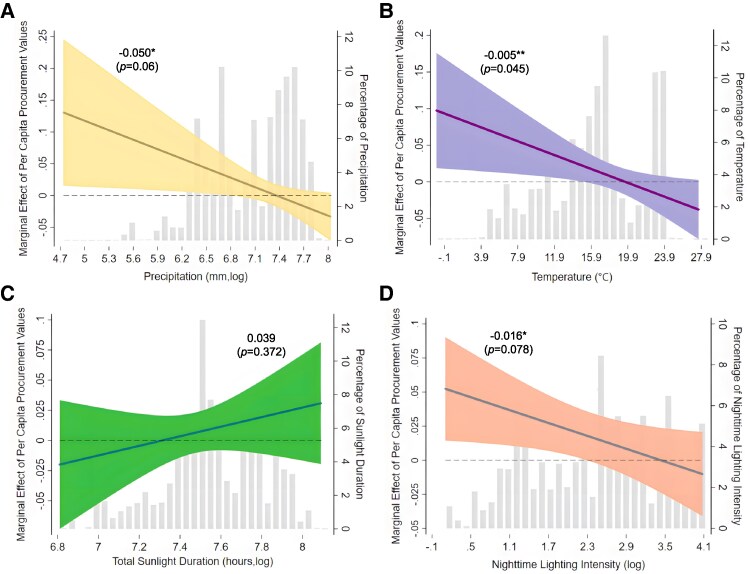
Heterogeneity analysis of urban climate and ambient lighting. A) Impact of surveillance cameras on migrants’ safety perception across varying levels of annual precipitation. B) Influence of surveillance cameras under different average temperatures. C) Effect of surveillance cameras under varying sunlight duration. D) Impact under different levels of nighttime lighting intensity. The *x*-axis shows the range of variation in the adjustment variables, while the *y*-axis on the left indicates the magnitude of the marginal effect. Statistical significance is represented by colored 90% CI zones. When the zone centered around the bold line does not cross zero on the left side *y*-axis (denoted by a horizontal line), the effect is significant at the 10% level. Annotated values above the colored zones represent interaction term coefficients, with *P*-values and asterisks indicating the significance of these interaction term coefficients. Additionally, histograms of precipitation, temperature, sunlight duration, and nighttime lighting intensity are shown in gray within the subplots, with the *y*-axes on the right indicating corresponding values. ***P<0.01, **P<0.05, *P<0.1.

Figure 4A shows that the interaction coefficient between precipitation and surveillance cameras is statistically significant and negative, indicating that excessive rainfall diminishes the positive effects of surveillance cameras on migrants’ safety perceptions. When annual average rainfall reaches ∼6.8 mm (*log*), the impact of surveillance cameras on migrants’ safety perceptions becomes negligible. Similarly, Fig. 4B reveals that as annual average temperature rises, the marginal benefits of surveillance cameras for migrants’ safety perceptions steadily decline. When the temperature reaches ∼14.9°C, the positive effect of surveillance cameras on migrants’ safety perceptions becomes insignificant.

Different types of urban ambient lighting, including sunlight and nighttime lighting, may also influence migrants’ perceptions of safety. Continuous exposure to sunlight appears to positively affect the sense of safety provided by surveillance cameras, although this effect is not statistically significant (Fig. 4C). In contrast, nighttime lighting and surveillance cameras act as substitutes in enhancing migrants’ safety perceptions (Fig. 4D). By illuminating the surrounding environment, city nighttime lighting increases the opportunity cost of criminal activities being detected, thereby serving as a deterrent to crime.

### Urban inclusiveness impacts migrants’ safety perceptions

The fear of crime, as noted by Pantazis (56), is shaped by social, environmental, and economic risks that introduce uncertainty in individuals’ ability to resist harm. In this context, the inclusiveness of host cities—spanning institutional, social, economic, and cultural dimensions—is crucial for shaping migrants’ perceptions of safety. Historically, China’s household registration (Hukou) system was a primary barrier to migrants (57, 58) (see Table S5). Although reforms have relaxed direct controls, the current points-based settlement system that built on educational attainment, occupational status, and social insurance contributions, has created human capital-based thresholds for urban settlement, excluding large segments of low-skilled and informally employed migrants. This exclusion deepens their socioeconomic vulnerabilities.

Here, we use the Hukou Registration Index (58) to assess the strictness of these settlement policies. Cultural integration constitutes an important dimension of urban inclusiveness and plays a crucial role in shaping migrants’ perceptions of safety. In particular, culturally exclusive environments, such as those dominated by clan-based social structures, can hinder migrants’ ability to integrate into the social fabric of the host city. These exclusionary settings, in turn, signal limited openness to newcomers and reinforce migrants’ feelings of marginalization, social distance, and perceived discrimination. Such experiences can heighten psychological vulnerability and contribute to a diminished sense of security. To capture the local strength of clan culture, we use the number of newly built clan temples as a proxy indicator (59, 60) to reflect the persistence and institutional visibility of traditional kinship networks. In this framework, stronger clan-based cultural dominance may serve as a barrier to inclusive urban environments and thus negatively affect migrants’ perceived safety. Furthermore, housing security is a key determinant of residents’ sense of belonging, yet high housing costs marginalize migrants both materially and psychologically—by limiting access to affordable accommodation, and by pricing many low-wage workers out of homeownership and long-term tenancy (61, 62). This erosion of social integration, together with persistent financial strain, may further amplify migrants’ perceptions of insecurity.

**Fig. 5.**
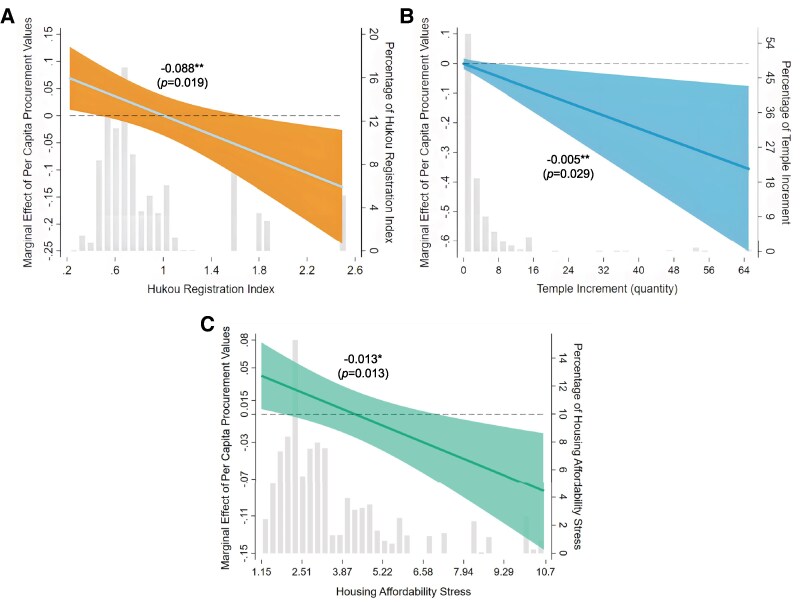
Heterogeneity analysis of Urban inclusiveness. A) Impact of surveillance cameras on migrants’ safety perception across varying levels of the Hukou registration index. B) Impact of surveillance cameras on migrants’ safety perception across varying levels of temple increment. C) Impact of surveillance cameras on migrants’ safety perception across varying levels of housing affordability stress. The *x*-axis shows the range of variation in the adjustment variables, and the *y*-axis on the left indicates the magnitude of the marginal effect. Statistical significance is represented by colored 90% CI zones. When the zone centered around the bold line does not cross zero on the left side *y*-axis (denoted by a horizontal line), the effect is significant at the 10% level. Annotated values above the colored zones represent interaction term coefficients, with *P*-values and asterisks indicating the significance of these interaction term coefficients. ***P<0.01, **P<0.05, *P<0.1.

Figure 5 shows the marginal effect plots of urban inclusiveness and surveillance cameras. Figure 5A shows that in cities with a higher Hukou registration index (indicating stricter qualifications for obtaining registration), the influence of surveillance cameras on migrants’ perceived safety is reduced. Specifically, when the Hukou registration index exceeds 0.6, the effect of surveillance cameras on migrants’ sense of security becomes insignificant. Figure 5B indicates that surveillance cameras have a significantly negative effect on perceived safety in cities with a large number of clan temples. Figure 5C indicates that in cities where housing cost burdens are higher, the positive effect of surveillance cameras on migrants’ perceived safety also declines. When the housing cost burden is below 2.17, surveillance cameras have a positive effect on migrants’ safety perceptions. However, once the burden exceeds 7.26, the effect turns negative, suggesting that excessive housing stress may undermine the reassuring function of surveillance infrastructure. This threshold is particularly relevant, as the national average housing cost burden in 2018 was ∼2.6, suggesting that in a substantial number of cities, the reassuring function of surveillance cameras may already be constrained by rising housing costs.

## Discussion

Safety, inclusiveness, and sustainability are critical in urban development. Community policing has long been regarded as an essential tool for deterring crime, as it enhances residents’ safety through rapid response and the establishment of police-community partnerships (63). Government-deployed surveillance cameras are similarly viewed as tools for crime prevention and maintaining public safety. However, our subgroup and interaction analyses reveal notable limitations in their effectiveness for improving migrants’ perceived safety. While these systems significantly reduce fear of crime among local residents, they have no comparable effect for migrants, underscoring divergent responses across groups. Although public attitudes toward surveillance in China are generally positive, this does not negate privacy concerns. On the contrary, it highlights the need for stronger privacy protections to sustain institutional trust.

It is essential to acknowledge that technology cannot replace intimate social relationships; rather, they complement one another in enhancing migrants’ perceived safety. Our work highlights the importance of geographic proximity in neighborhood social relations for strengthening migrants’ sense of safety. Informal social support, such as timely assistance from neighbors, significantly improves migrants’ perceptions of safety.

We also show that migrants’ sense of safety is shaped not only by the potential risk of crime but also by their ability to mitigate these risks. Extreme weather conditions, such as high temperatures and excessive precipitation, can contribute to an increase in regional crime rates and diminish the positive effects of surveillance cameras on migrants’ safety perceptions. Additionally, nighttime lighting plays a crucial role in enhancing migrants’ sense of safety. Besides, the results on urban inclusiveness heterogeneity point out that migrants’ apprehension of criminal behavior is connected to a sense of insecurity stemming from institutional marginalization. These findings highlight the need for a public safety strategy that reflects the layered and varied nature of safety perceptions among different social groups. In particular, improving migrant integration through equitable access to urban services, housing security, and inclusive neighborhood-building is essential to fostering both their actual and perceived safety.

Our study further investigates the interaction between surveillance cameras and community-based initiatives in crime prevention and safety awareness, an area that remains under explored. While surveillance cameras can enhance residents’ safety perceptions by deterring crime, technological advancements do not diminish the essential role of human relationships. Trust and support from close social connections remain critical sources of individuals’ sense of safety. Integrating digital surveillance technology with intimate social networks can provide greater benefits for migrants’ sense of security. This reflects a shift in governance, where citizens are not merely passive subjects but active participants in the process. A collaborative network, comprising citizen participation, government cooperation, and technological assistance, plays a crucial role in urban management.

Moreover, our analysis highlights the importance of environmental protection to mitigate the adverse social effects of extreme weather. Enhancing nighttime lighting is also vital for improving migrants’ safety perceptions. Finally, building cities that provide equal opportunities, shared resources, and a sustainable, inclusive environment can significantly increase the resilience of marginalized migrants to risks.

Some limitations of this study should be addressed in future research. First, as the study focuses on urban areas, our findings may disproportionately represent individuals with higher levels of education and economic status. Future research should aim to include larger and more diverse samples, for example, with particular attention to rural populations and low-income groups. Second, our analysis is based on mixed cross-sectional data, highlighting the need for longitudinal data to better understand the causal relationships between surveillance technology, social ties, and perceived safety. Third, our findings mainly focus on the pre-COVID-19 pandemic context. Applying these results to the postpandemic era may overestimate the protective role of surveillance, particularly among socially vulnerable groups like migrants, as the pandemic likely reshaped public trust, social ties, and surveillance attitudes in China—changes that merit further research.

## Materials and methods

### Data sources

Our data on migrants comes from the two waves of the China Labor Force Dynamic Survey (CLDS) (42), conducted by Sun Yat-sen University in 2016 and 2018. This dataset includes a wide range of variables such as individual safety perceptions, migration experiences, social ties, and demographic characteristics. Given the comparatively underdeveloped surveillance camera infrastructure in rural areas, we limited our sample collection to urban areas. The distinction between migrants and local residents is based on individuals aged 14 or older who have experienced mobility for 6 months or more (64). Additionally, outliers in the social network variables were removed. In total, we retained 11,411 interviewees, comprising 3,949 migrants and 8,462 local residents.

The surveillance cameras data are collected from the China Government Procurement website (https://www.ccgp.gov.cn), which is publicly accessible. We identified keywords related to surveillance cameras based on the “Government Procurement Category Classification Catalog.” These keywords include surveillance cameras, alarm sensors, digital video recorders, video splitters, monitoring video walls (tiled display monitors), monitors, access control systems, and others (see Fig. S1). To minimize sample selection bias stemming from the higher upload rate of government procurement contracts after 2013, we extracted all government procurement bidding and transaction announcements from 2013 to 2017 (65). A total of 156,833 announcements were collected.

We refined this dataset by removing void and failed bids, as well as null values. Additionally, we eliminated duplicates based on contract names, procurement dates, and other relevant details. From this dataset, we finally identified 22,572 announcements where the buyer was identified as police (Public Security), including contracts procured by public security bureaus, sub-bureaus, and police stations. We then applied a semisupervised named entity recognition algorithm (66) in combination with manual verification to determine each contract’s value and the buyer’s location.

### Model setting

We use classic OLS model to examine the correlation between surveillance cameras and residents’ perceived safety. Our empirical analysis begins by specifying the following equation:


(1) $$\begin{array}{rl} Safety_{i,t} & =\alpha_{0}+\alpha_{1}ln(Cameras_{c,t-1})+\alpha_{i}\;Controls_{c,t-1}\\ & \;+\sigma_{\;p(c)\times t}+\epsilon_{i,t} \end{array}$$

where $Safety_{i,t}$ represents the safety perception of individual *i* in year *t*, and $\ln\;(Cameras_{c,t-1})$ is the logarithm of the accumulated per capita procurement value of surveillance cameras in city *c* during year t−1. *Controls* denotes a series of control variables at the individual, household, community, and city levels. $\sigma_{\;p(c)\times t}$ refers to the fixed effects for province *p*, where city *c* is located, and year t−1. Finally, $\epsilon_{i,t}$ represents the error term.

### Main variables

Safety perception. Community safety perception is used as a proxy for residents’ overall safety perception. In China, communities encompass a wide range of areas, including residential zones, parks, schools, and commercial facilities. The size of these communities varies, ranging from a few blocks to entire small towns, with populations ranging from several thousand to tens of thousands. The CLDS assesses individuals’ subjective sense of safety within their community through the question: “Do you feel that your community is safe?” Responses are recorded on an ordinal scale from 1 to 4, corresponding to: very safe, relatively safe, not very safe, and very unsafe. We recoded these responses from 1 to 4, with higher scores reflecting a greater sense of individual safety.

Surveillance cameras. Given that surveillance cameras typically remain operational for several years, we use the per capita cumulative value of surveillance cameras procured in prefecture-level cities from the previous year (log-transformed) as a measure of the spatial distribution of video surveillance. This metric effectively reflects the level of surveillance camera development in the local area.

Social ties. We measure emotional local social ties using two variables: “the number of people with close local relationships” and “the number of people in whom respondents can confide within these close relationships.” Instrumental local social ties are measured by “the number of people locally who can lend 5,000 CNY.” Neighborhood social ties are assessed through two variables: “the level of familiarity with neighbors in the community (1–5),” where the ordinal level goes from 1 (lowest) to 5 (highest); and “the degree of trust toward neighbors in the community (1–5),” where the ordinal level goes from 1 (lowest) to 5 (highest). To address the impact of outliers, we apply a 5% two-sided winsorization to local social ties data. Additionally, we excluded samples where the number of people confided in exceeded the total number of close local relationships to ensure the logical consistency of the local social ties measurements.

Control variables. Prior research has shown that individual, family, and community characteristics can influence residents’ perceived safety (26, 67, 68). At the individual level, we control for factors such as gender, age, marital status, years of education, socioeconomic status, height, weight, and physical appearance. At the family level, we include controls for car ownership, number of siblings, and pollution levels. At the community level, we account for local environmental conditions such as pollution levels. Additionally, city-level characteristics, which may also affect residents’ perceptions of safety, are accounted for in the analysis (69). A more detailed explanation of the rationale behind the selection of control variables is provided in SI Appendix S8.

Instrumental and heterogeneity variables. The data on collective protest events in China, collected in real-time from social media by Zhang and Pan (70), include over 100,000 incidents from 2010 to 2017. These events encompass activities such as boycotts, demonstrations, marches, sit-ins, and strikes. In contrast, crimes that influence residents’ perceived safety typically involve incidents like murder, robbery, and burglary, where the primary targets are the physical safety or property of residents (71). Collective protest events, however, tend to target political or economic authorities and exhibit an element of randomness. To ensure the validity of the exclusion restriction, we exclude data from years close to the survey period. Instead, we calculate the ratio of the increment in collective protest events in each Chinese city in 2013 to the stock of events in the same year.

In Table S6, we present the definition of the above variables and their descriptive statistics. We also include the questionnaire items used in the study from the original CLDS, along with their translations, in Table S7. We sourced climate data, including temperature, precipitation, and sunshine duration, from the National Centers for Environmental Information (NCEI, https://www.ncei.noaa.gov) under the National Oceanic and Atmospheric Administration (NOAA, https://www.noaa.gov). Additionally, nighttime lighting data were obtained from the National Geophysical Data Center (NGDC, https://www.ngdc.noaa.gov), also part of NOAA. To calculate the average nighttime lighting intensity for cities, we aggregated the corrected nighttime lighting raster data by administrative boundaries and divided the total by the land area of each administrative region.

The annual increase in ancestral temples in prefecture-level cities across the country was used as a metric to gauge the local level of clan culture (72).

## Supplementary Material

pgaf331_Supplementary_Data

## Data Availability

The China Labor-force Dynamics Survey (CLDS) data we used and the surveillance camera data are available at https://figshare.com/s/0080a0068cf0e1e85548. Data from the China Urban Statistical Yearbook is publicly available from the National Bureau of Statistics of China (https://data.stats.gov.cn/english/). The nighttime lighting data are publicly available at: https://dataverse.harvard.edu/dataset.xhtml?persistentId=doi:10.7910/DVN/YGIVCD. Code used to produce Fig. 2 in the analysis has been made available in the Supplementary Information.
