# Supplementary material for: The heterogeneous impact of public security cameras on safety perceptions in cities: Evidence from China
Source: PNAS Nexus. 2025 Oct 16;4(10):pgaf331. doi: 10.1093/pnasnexus/pgaf331 (PMC12575433; doi:10.1093/pnasnexus/pgaf331)
Supplement: pgaf331_Supplementary_Data [file pgaf331_supplementary_data.zip › PNASNEXUS-PNASNEXUS-2025-00303-TR-s09.pdf]

PNAS nexus

Supporting Information for

The Heterogeneous Impact of Public Security Cameras on Safety Perceptions in Cities: Evidence from China

Pinghan Liang, Yadi Liu, Yuchen Guo, Fanqi Zeng

Email: liangph5@mail.sysu.edu.cn (P.L.), fanqi.zeng@sociology.ox.ac.uk (F.Z.)

This PDF file includes:

Figures S1 to S2
Tables S1 to S7

1. Supplementary Table S1

Results for heterogeneity of institutional trust

Variable names	Safety perception	Safety perception
	High_ Institutional trust	Low_ Institutional trust
	(1)	(2)
Ln(Per capita procurement value)	0.019***	-0.015
	(0.006)	(0.026)
Individual characteristics	√	√
Family characteristics	√	√
Community characteristics	√	√
City characteristics	√	√
Year*Province	√	√
Empirical p-value	0.007**	
N	7,164	2,459
adj. R-squared	0.086	0.065

Note: Column (1) presents the regression results for cities with high levels of institutional trust, while Column (2) shows the results for cities with low levels of institutional trust. The data on institutional trust is derived from the 2016 China Family Panel Studies (CFPS). *$p < 0.1$, **$p < 0.05$, ***$p < 0.01$.

2. Supplementary Table S2

Results for endogeneity test

Variable names	Safety perception	Safety perception
	All sample	All sample
	(1)	(2)
	Second-stage regression	Second-stage regression
ln(Per capita procurement value)	0.196***	0.200***
	(0.024)	(0.024)
Migrants		0.971***
		(0.15)
Cameras*Migrants		-0.075***
		(0.011)
Individual characteristics	√	√
Family characteristics	√	√
Community characteristics	√	√
City characteristics	√	√
	First-stage regression	First-stage regression
Collective Protest Events(IV)	3.868***	3.703***
	(0.286)	(0.267)
Individual characteristics	√	√
Family characteristics	√	√
Community characteristics	√	√
City characteristics	√	√
Year*Province	√	√
F-stat.	182.250	191.122
N	9,629	9,629
adj. R-squared	0.740	0.766

Note: Columns (1) and (2) report the regression estimates using Two-Stage Least Squares (2SLS), with previous collective protest events serving as the instrumental variable. Standard errors are clustered by individuals. $^{*}p < 0.1$, $^{**}p < 0.05$, $^{***}p < 0.01$.

3. Supplementary Table S3.1

Results for robustness checks (1)

Variable names	Safety perception	Safety perception	Safety perception	Safety perception	Safety perception
	All sample	All sample	Migrants	Local	All sample
	(1)	(2)	(3)	(4)	(5)
Ln(Per capita procurement value)	0.019***	0.020***	-0.004	0.027***	0.021***
	(0.005)	(0.005)	(0.012)	(0.005)	(0.005)
Migrants		0.077			0.102
		(0.075)			(0.098)
Procurement value*Migrants		-0.010*			-0.012*
		(0.006)			(0.007)
Individual characteristics	√	√	√	√	√
Family characteristics	√	√	√	√	√
Community characteristics	√	√	√	√	√
City characteristics	√	√	√	√	√
Year*Province	√	√	√	√	√
N	9,569	9,569	1534	8093	9628
adj. R-squared	0.069	0.070	0.043	0.076	0.071

Note: Columns (1) and (2) present results after excluding individuals who moved to the city within the past six months. Columns (3) to (5) use the consistency between an individual's registered residence and the surveyed location to identify migrants. Specifically, Columns (3) and (4) show subgroup analysis for migrants and local residents, while Column (5) further reports results from interaction terms. All coefficients are estimated using the OLS model. Standard errors are clustered by individuals. *$p < 0.1$, **$p < 0.05$, ***$p < 0.01$.

4. Supplementary Table S3.2

Results for robustness checks (2)

Variable names	Safety perception	Safety perception	Safety perception	Mean_Safety perception
	All sample	All sample	All sample	All sample
	(1)	(2)	(3)	(4)
Ln(Per capita procurement value)	0.012***	0.021***	0.025***	0.020***
	(0.004)	(0.005)	(0.005)	(0.002)
Migrants		0.080		
		(0.074)		
Procurement value*Migrants		-0.010*		
		(0.006)		
Crime Rate	-	-	√	-
Individual characteristics	√	√	√	-
Family characteristics	√	√	√	-
Community characteristics	√	√	√	-
City characteristics	√	√	√	√
Year*Province	√	√	√	√
N	8,952	8,622	8,622	9,904
adj. R-squared	0.042	0.074	0.074	0.444

Note: Columns (1) and (2) present results after excluding the four municipalities. In Column (3), crime rates are additionally controlled for. In Column (4), we calculate the average perceived safety of all respondents in a city to measure the overall perception of safety. All coefficients are estimated using the OLS model. Standard errors are clustered by individuals. *$p < 0.1$, **$p < 0.05$, ***$p < 0.01$.

5. Supplementary Table S4

Variable names	Locals			Migrants			
	N	Mean	Std.Dev.	N	Mean	Std.Dev.	*P*-value
Fear of walking alone at night	3,688	1.912	1.057	1,122	1.953	1.088	0.126
Fear of home burglary	3,688	2.302	1.174	1,122	2.419	1.213	0.002**
Fear of being targeted for displaying wealth.	3,688	2.126	1.151	1,122	2.174	1.179	0.113

Note: Data on fear of crime is drawn from the 2018 wave of the China Labor-force Dynamics Survey (CLDS). A two-sided *t*-test is used to assess mean differences in fear of crime between local residents and migrants. *$p < 0.1$, **$p < 0.05$, ***$p < 0.01$.

6. Supplementary Table S5

Hukou Registration Index for Sample Cities (Project Pursuit Model)

City	Investment	Home purchase	Talent program	Employment	Composite Index
2000-2013					
Beijing	0.5888	0.2691	0.2695	0.6411	0.8983
Shanghai	0.2449	0.3363	0.1788	0.5989	0.6834
Guangzhou	0.2057	0.2354	0.3705	0.4734	0.4867
Shenzhen	0.4188	0.3363	0.3942	0.1123	0.2790
Tianjin	0.1849	0.2354	0.3705	0.1123	0.1697
Hangzhou	0.0566	0.1110	0.0360	0.0122	0.0875
Nanjing	0.0421	0.0444	0.0460	0.1139	0.1563
Jinan	0.1712	0.0666	0.0190	0.0236	0.1226
Chongqing	0.0711	0.0445	0.0161	0.0572	0.1042
Qingdao	0.0552	0.0889	0.0661	0.0348	0.1040
Dalian	0.0566	0.0003	0.1162	0.0348	0.0936
Ningbo	0.0552	0.0003	0.0460	0.0124	0.0765
Xiamen	0.0421	0.0003	0.1766	0.0912	0.2050
Chengdu	0.0711	0.0667	0.0921	0.1478	0.2027
Wuhan	0.0421	0.0445	0.0962	0.0349	0.1254
Harbin	0.0276	0.0223	0.0159	0.0123	0.0520
Shenyang	0.0160	0.0223	0.0460	0.0571	0.0779
Xi'an	0.0421	0.0223	0.0862	0.0461	0.1040
Changchun	0.0189	0.0444	0.0661	0.0235	0.0943
Changsha	0.0566	0.0003	0.046	0.1707	0.1682
Fuzhou	0.0552	0.0002	0.0661	0.1927	0.2469
Zhengzhou	0.0552	0.0223	0.046	0.0235	0.1023
2014-2016					
Beijing	0.5717	0.2582	0.2633	0.7692	1.0729
Shanghai	0.1937	0.3730	0.2375	0.6242	0.8182
Guangzhou	0.2435	0.1443	0.3909	0.2471	0.6461
Shenzhen	0.4291	0.3730	0.2633	0.1592	0.6461
Tianjin	0.1978	0.2582	0.4313	0.1592	0.5108
Hangzhou	0.0492	0.0909	0.0559	0.0281	0.1269

Nanjing	0.0372	0.0363	0.0486	0.0542	0.1122
Jinan	0.0127	0.0364	0.0357	0.0825	0.1109
Chongqing	0.0745	0.0364	0.0348	0.1624	0.1628
Qingdao	0.0492	0.0364	0.0615	0.0340	0.1093
Dalian	0.0543	0.0909	0.0357	0.0366	0.1109
Ningbo	0.0372	0.0364	0.0486	0.1393	0.1825
Xiamen	0.0372	0.0182	0.1264	0.1277	0.1825
Chengdu	0.0625	0.0364	0.0745	0.0549	0.1294
Wuhan	0.0492	0.0364	0.0745	0.0633	0.1269
Harbin	0.0366	0.0182	0.0430	0.0164	0.0707
Shenyang	0.0265	0.0182	0.0486	0.0469	0.0825
Xi’an	0.0372	0.0182	0.0479	0.1118	0.1269
Changchun	0.0170	0.0363	0.0615	0.0470	0.0940
Changsha	0.0499	0.0182	0.0486	0.0352	0.082
Fuzhou	0.0203	0.0001	0.1386	0.1118	0.1825
Zhengzhou	0.0170	0.0182	0.0486	0.0352	0.0820

Note: Adapted from “Appendix 3. Additional Results on the Hukou Registration Index” in Zhang, J., Wang, R., & Lu, C. (2019). A quantitative analysis of Hukou reform in Chinese cities: 2000–2016. Growth and Change, 50(1), 201-221.

7. Supplementary Table S6

Definition of variables and descriptive statistics

Variable names	N	Mean	Std.Dev.	Min	Max	Definition
Panel A: Main variables						
Safety perception	11,375	3.072	0.621	1.000	4.000	Categorical: Do you feel safe in your community? (1=Very unsafe, ..., 4=Very safe)
Per capita procurement value on surveillance cameras (log)	10,007	12.912	2.544	4.916	18.392	The logarithm of the cumulative per capita purchase value in prefecture-level cities (CNY per 10,000).
Panel B: Individual, community, and city-level control variables						
Age	11,375	42.907	13.43	15.000	64.000	Respondent's age distribution: 15-30 years old,21.91%; 31-45 years old, 30.47%; 46-64 years old, 47.62%.
Gender	11,375	0.455	0.498	0.000	1.000	Binary: Female=0 (54.52%); Male=1 (45.48%).
Education	11,354	10.965	3.943	0.000	22.000	Respondent's level of education. Proportions: 0-9 years, 45.83%; 10-16 years, 52.66%; >16 years,1.51%.
Marriage	11,375	0.788	0.409	0.000	1.000	Binary: Unmarried=0 (21.20%); Cohabiting, First marriage, Remarriage=1 (78.80%).
Height	11,375	164.817	7.985	123.000	195.000	Respondent's height (cm).
Weight	11,375	61.722	12.192	22.000	132.000	Respondent's weight (kg).
Appearance	11,372	6.596	1.549	1.000	10.000	Categorical: Evaluation of the respondent's appearance (Rating from 1-10, from lowest to highest)
Health	11,370	3.781	0.905	1.000	5.000	Categorical：Respondent's self-assessment of their health condition (1=Very healthy, ..., 5=Very unhealthy)
Perceived Social Status	11,375	4.560	1.748	1.000	10.000	Categorical：Respondent's self-

						assessment of their current social status (Rating from 1-10, from lowest to highest)
Number of siblings	11,375	2.524	2.009	0.000	13.000	Number of siblings of the respondents
Car	11,368	0.350	0.477	0.000	1.000	Binary: Whether the respondent's household owns a car (No=0, 65.02%; Yes=1,34.98%)
Per capita GDP	11,112	9.351	9.010	0.188	51.701	The ratio of the city's gross domestic product (GDP) to the total population at the end of the previous year (One Hundred Thousand).
Government size	11,273	0.128	0.071	0.014	0.513	The ratio of the city's general public budget expenditure to its gross domestic product (GDP) in the previous year.
Unemployment Rate	11,375	0.049	0.036	0.004	0.264	The ratio of the registered urban unemployed population to the total of urban unemployed and employed population in the previous year (%).
Numbers of Internet broadband subscribers	11,375	21.961	21.365	1.000	120.500	Number of internet broadband subscribers in the previous year (One Hundred Thousand).
Community pollution	11,074	0.224	0.417	0.000	1.000	Binary: No Pollution=0 (77.60%), Pollution Present=1 (22.40%).
Number of crimes	10,321	1.914	1.294	0.103	5.241	Cumulative Number of Urban Crimes,1985 - 2015 (in ten thousand)
Panel C: Instrumental and heterogeneous variables						
Collective protest events*	11,375	0.493	0.126	0.071	0.925	The ratio of the increment in collective protest events in 2013 to the cumulative events in the same year.
Temperature#	11,375	16.445	5.204	0.548	24.442	Annual average temperature where the individual resides.

Precipitation(log)[#]	11,375	7.060	0.524	5.537	7.976	Annual average Precipitation where the individual resides.
Sunshine duration(log) [#]	11,375	7.565	0.239	6.810	8.012	Total annual sunshine duration where the individual resides.
Nighttime lighting intensity (log) [#]	11,375	2.451	0.988	0.158	4.086	The corrected average nighttime light intensity per unit of administrative area in the city where the individual resides.
Hukou registration[#]	7,532	0.920	0.534	0.223	2.496	The strictness of migrants to get local urban Hukou.
Clan culture[#]	10,622	3.380	7.920	0.000	65.000	The increment of ancestral temples in the city that year.
Housing affordability stress[#]	1,0159	3.548	2.088	1.177	10.649	The ratio of average housing price (CNY /m²) to average monthly income (CNY /month) in the city where the individual resides.

[a] Data source: Authors' own calculation based on CLDS (2016, 2018).
[b] Variables marked * are used as instrumental variable.
[c] Variables marked # are used for heterogeneity testing.

8. Supplementary Table S7: CLDS Questionnaire Items

Chinese Questions	English Translation of Questions (note: translated by the authors of the paper)
**个人安全感题目**	**Safety perception**
你觉得你所在的社区安全吗？<br>1. 很安全 2. 较安全 3. 不太安全 4. 很不安全	Do you feel that your community is safe?<br>1. Very safe 2. Relatively safe 3. Not very safe 4. Very unsafe
**社会网络相关题目**	**Social Network**
在本地，您有多少关系密切，可以得到他们支持和帮助的朋友/熟人？	In your local area, how many close friends or acquaintances do you have who can provide support and help?
在本地这些关系密切的人中，您可以向他/她诉说心事的有几个？	Among these close contacts, how many can you confide in when you need to share personal matters?
在本地这些关系密切的人中，您可以向他/她借钱（5000 元为标准）的有几个？	Among these close contacts, how many could you borrow money from (with RMB 5,000 as the reference)?
您对本社区的邻里、街坊及其他居民信任吗？	Do you trust your neighbors and other residents in your community?
您与本社区的邻里、街坊及其他居民互相之间有互助吗？<br>1. 非常少 2. 比较少 3. 一般 4. 比较多 5. 非常多	To what extent is there mutual assistance among neighbors and residents in your community?<br>1. Very limited 2. Relatively limited 3. Moderate 4. Relatively much 5. Very much
**犯罪恐惧感相关题目**	**Fear of Crime**
夜晚独自外出会有安全风险<br>1. 非常不同意 2. 不同意 3. 一般 4. 同意 5. 非常同意 99998. 不知道	Going out alone at night poses safety risks<br>1. Strongly disagree 2. Disagree 3. Neutral 4. Agree 5. Strongly agree 99998. Don’t know
不锁好门窗会有被入室盗窃的风险<br>1. 非常不同意 2. 不同意 3. 一般 4. 同意 5. 非常同意 99998. 不知道	There is a risk of burglary if doors and windows are not properly locked<br>1. Strongly disagree 2. Disagree 3. Neutral 4. Agree 5. Strongly agree 99998. Don’t know
露财会有被盯上的风险<br>1. 非常不同意 2. 不同意 3. 一般 4. 同意 5. 非常同意 99998. 不知道	Displaying wealth may increase the risk of being targeted<br>1. Strongly disagree 2. Disagree 3. Neutral 4. Agree 5. Strongly agree 99998. Don’t know
**流动人口识别题目**	**Migrant Identification**
请问您 14 岁以来，您是否有过跨县市迁移经历？<br>1. 是 2. 否	Since the age of 14, have you ever experienced migration across counties/cities?<br>1. Yes 2. No
您的户口是在：<br>1. 本村/居委会 2. 本乡镇（街道）其他村居委会 3. 本县（县级市、区）其他乡镇街道 4. 本县区以外	Where is your household registration (hukou) located<br>1. This village/neighborhood committee 2. Another village/neighborhood committee in this township/sub-district<br>3. Another township/sub-district in this county (county-level city, district) 4. Outside this county/district

控制变量相关题目	Control Variables
您的出生年月是	What is your date of birth?
您正确的婚姻状况是<br>1. 未婚 2. 初婚 3. 再婚 4. 离异 5. 丧偶 6. 同居	What is your current marital status?<br>1. Never married 2. First marriage 3. Remarried 4. Divorced 5. Widowed 6. Cohabiting
您有几位兄弟姐妹?	How many siblings do you have?
身高 ____ 厘米	Height: ____ cm
体重 ____ 斤	Weight: ____ kg
您认为自己现在的健康状况如何?<br>1. 非常健康 2. 健康 3. 一般 4. 比较不健康 5. 非常不健康	How would you rate your current health status?<br>1. Very healthy 2. Healthy 3. Fair 4. Unhealthy 5. Very unhealthy
我们的社会里，有些人居于顶层，有些人则在底层。下面这种卡片上有一个从上往下的梯子，最高的“10”分代表最顶层，最低的“1”分代表最底层。您认为您自己目前在哪个等级上?	In our society, some people are at the top while others are at the bottom. Here is a ladder with steps from 1 to 10, where “10” represents the top and “1” the bottom. Which step do you think you are currently on?
您的最高学历是：______	What is your highest level of education?
您觉得被访者的长相怎样?（长相越好，评分越高）1－10 分	How would you rate the respondent’s appearance? (The better the appearance, the higher the score) 1–10
您家里是否有汽车?<br>1. 否 2. 是	Does your household own a car?<br>1. No 2. Yes
本社区行政范围内是否有环境污染?<br>1. 是 2. 否	Within the administrative boundary of your community, is there environmental pollution?<br>1. Yes 2. No

9. Supplementary S8

Supplementary S8.1: Measurement of Surveillance Procurement Intensity

To measure the intensity of surveillance procurement at the prefecture level, we use the per capita cumulative procurement value of surveillance cameras equipment (CNY per 10,000) up to the year prior to the survey. Compared to the number of surveillance procurement contracts, the total procurement value provides a more accurate reflection of the significant variation in local government investment, which in turn indicates differences in local government priorities and attitudes toward surveillance infrastructure. Surveillance cameras are not standalone devices but are typically part of integrated systems. Therefore, our procurement data includes not only surveillance cameras but also related components such as sensors and facial recognition systems. Specifically, we calculate the total value of video surveillance procurement in a city and divide it by the total population at the end of the year. The logarithm of this per capita procurement value is used as the main independent variable. Population data are obtained from *the China Urban Statistical Yearbook.*

Supplementary S8.2: Named Entity Extraction from Procurement Texts

This study applies a semi-supervised named entity recognition (NER) algorithm to process surveillance equipment procurement data from government contracts, which total over one million entries. The approach addresses two core challenges: limited labeled data and the complexity of unstructured procurement text. We began by manually labeling a small set of representative samples, and then used pseudo-labeling and consistency regularization to train the model on unlabeled data. This process reduced manual annotation workload by more than 70 percent.

The algorithm is well suited to the structure of procurement documents, which often feature varied expressions for key entities, such as contract value ("contract amount," "total cost"), and domain-specific terms like "sensor" or "monitoring system." Through domain-adaptive fine-tuning, the model was able to recognize entities with high accuracy. To handle entity ambiguity, it also leveraged frequent co-occurrence patterns to detect entity boundaries and resolve cases such as abbreviated place names and variations in agency titles. To ensure the accuracy of extracted information, particularly contract values and buyer locations, all identified entities were manually verified following automated processing.

Supplementary S8.3: Control Variables and Justification

We include a comprehensive set of control variables across four levels—individual, family, community, and city—each of which captures important sources of heterogeneity that may shape migrants' perceptions of safety.

At the **individual level**, we account for factors related to physical and social vulnerability that influence personal security perceptions. Gender is controlled for due to the well-documented gendered fear of crime, particularly among women (1). Health status reflects perceived physical resilience and dependence on external protections (2). Age captures differences in physical capability, cognitive development, and routine activity patterns that influence how individuals evaluate safety infrastructure (3). Marital status is included as a proxy for emotional and economic support, which can affect perceived security (4). Education level influences access to information and attitudes toward surveillance technologies (5). Socioeconomic status reflects both exposure to risk and divergent views on public surveillance, while physical characteristics (e.g., height, weight, appearance) are known to shape perceived victimization risks and, hence, trust in protective infrastructure (6).

At the **family level**, we control for the number of siblings, which captures the size of an individual's informal support network (7). Those with more siblings may rely less on formal surveillance for safety assurance. Car ownership serves as a proxy for both economic security and mobility; greater access to private transportation reduces exposure to perceived unsafe environments, thus influencing perceived safety (8).

At the **community level**, we include environmental pollution as a visible indicator of neighborhood disorder and social neglect, both of which heighten fear of crime (9). Pollution is also correlated with community-level socioeconomic disadvantage, which has been empirically linked to higher crime rates and diminished feelings of safety among residents.

At the **city level**, we control for internet broadband access, as exposure to online content can amplify crime-related fears or shift attitudes toward surveillance (10). The unemployment rate is included due to its strong empirical association with local crime prevalence (11). We also control for government size, which reflects the administrative capacity to invest in public safety infrastructure (12), and per capita GDP, which serves as an indicator of overall economic development and its associated effects on both crime rates and public safety spending (13).

10. Supplementary Figure S1

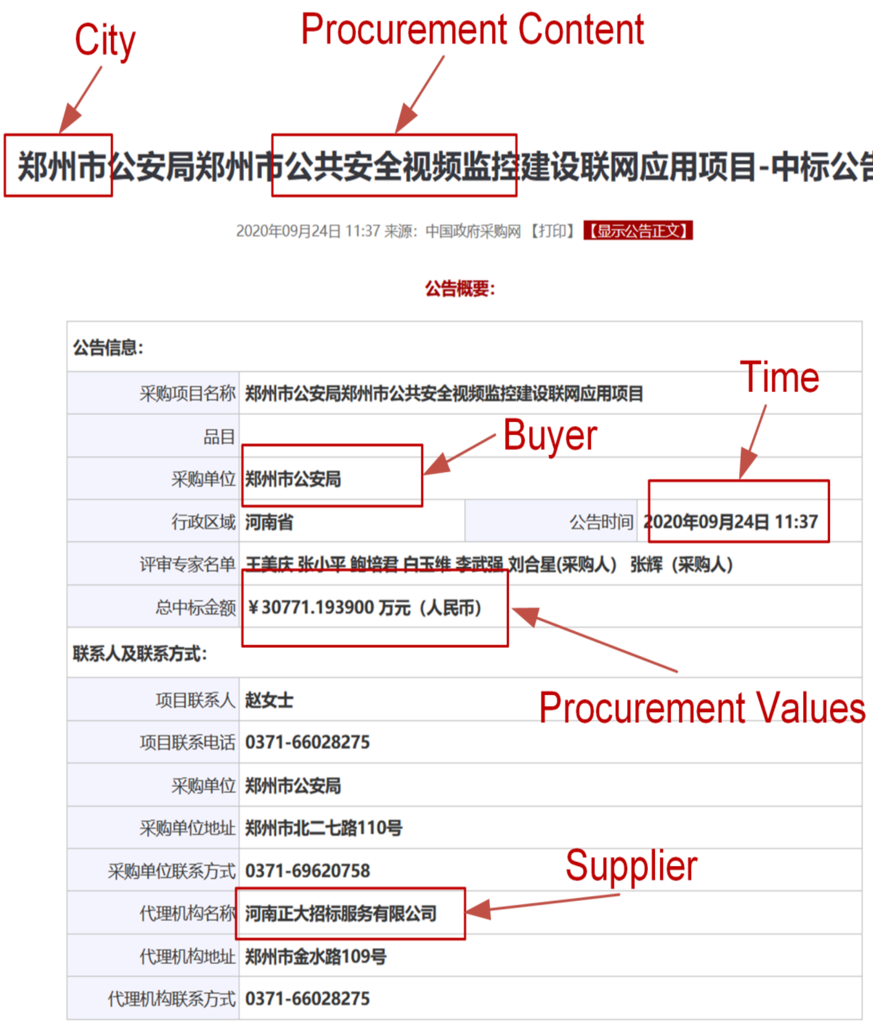

The government procurement announcement from local governments in China serves as an illustrative source for extracting data on city surveillance cameras. We match cities based on the address of the purchasing entity, with a primary focus on public security departments, and align this with the cities where CLDS respondents are located. This announcement provides comprehensive details on surveillance camera procurement, including key information such as the buyer, supplier, procurement date, procurement values, and procurement content.

11. Supplementary Figure S2

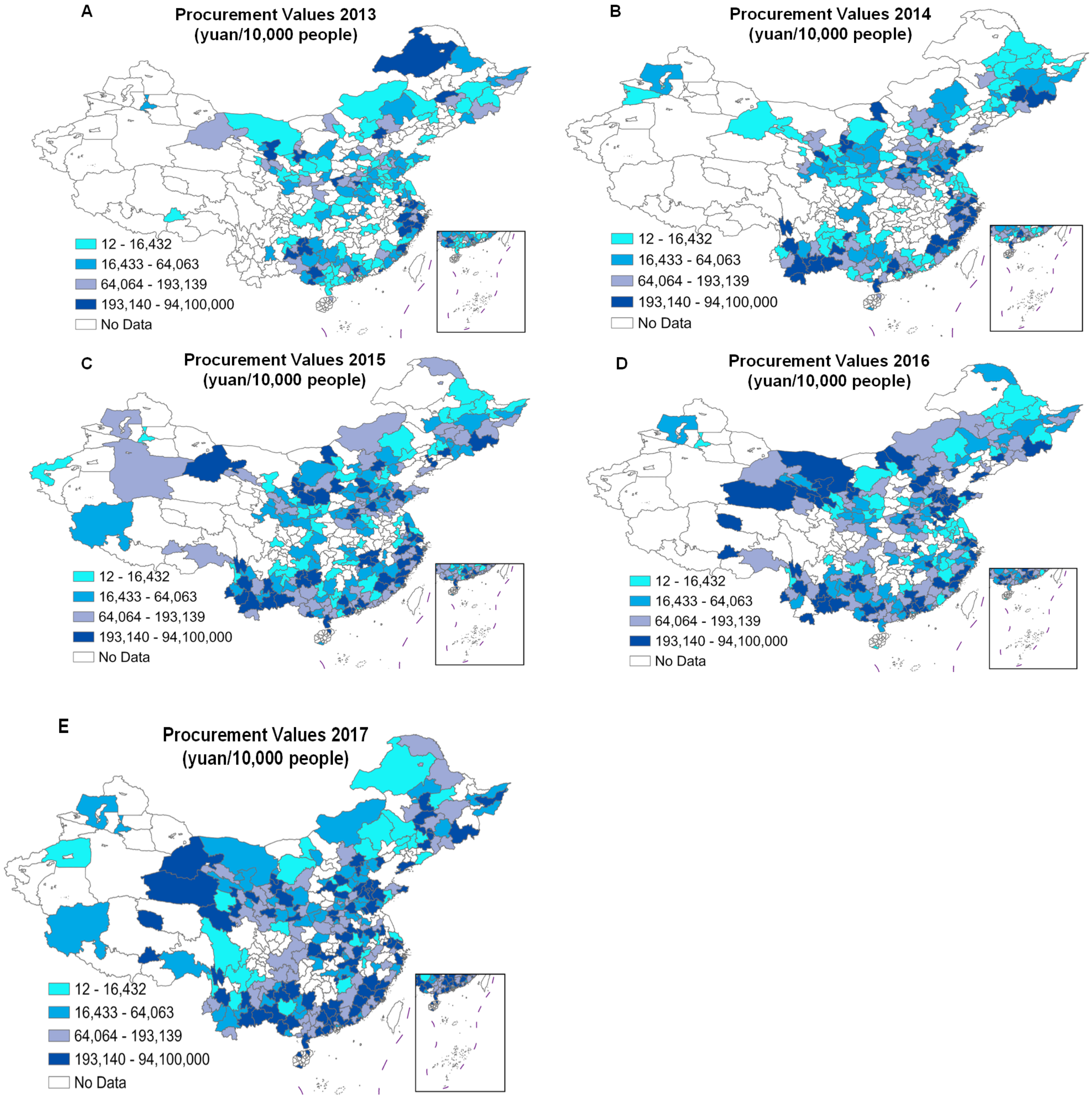

Yearly distribution map of total procurement value for surveillance cameras (2013-2017). (**A**) Procurement Expenditure for City Surveillance Cameras in 2013. (**B**) Procurement Expenditure for City Surveillance Cameras in 2014. (**C**) Procurement Expenditure for City Surveillance Cameras in 2015. (**D**) Procurement Expenditure for City Surveillance Cameras in 2016. (**E**) Procurement Expenditure for City Surveillance Cameras in 2017. The legend reflects changes in procurement amounts for city surveillance cameras over the years. Blue areas indicate cities where surveillance cameras were procured, with darker shades representing higher procurement values. The sample size of cities was 187 in 2013, 221 in 2014, 243 in 2015, 250 in 2016, and 256 in 2017.

Reference

1.EA Yeater, TA Treat, RJ Viken, AD Bryan, Risk processing and college women’s risk for sexual victimization. Psychol. Violence 10, 575–583 (2020).

2.J Jackson, M Stafford, Public health and fear of crime: A prospective cohort study. Br. J. *Criminol*. **49**, 832–847 (2009).

3.C Hale, Fear of crime: A review of the literature. *Int. Rev. Victim*ol. **4**, 79–150 (1996).

4.J Zhu, Z Li, Inequality and crime in China. Front. *Econ. China* **12**, 309–339 (2017).

5.AM Maineri, P Achterberg, R Luijkx, Switch on the Big Brother: Investigating the educational gradients in acceptance of online and public areas surveillance among European citizens. *Eur. Soc.* **24**, 628–656 (2022)

6.J Savolainen, JR Brauer, N Ellonen, Beauty is in the eye of the offender: Physical attractiveness and adolescent victimization. *J. Crim. Justice* **66**, 101652 (2020).

7.CE Ross, SJ Jang, Neighborhood disorder, fear, and mistrust: The buffering role of social ties with neighbors. *Am. J. Community Psychol.* **28**, 401–420 (2000).

8.Y Zhang, JD Fricker, Y Li, The impact of personal vehicle ownership on perceived accessibility and safety: Evidence from China. *Sustainability* **12**, 3563 (2020).

9.J Burkhardt, et al., The effect of pollution on crime: Evidence from data on particulate matter and ozone. *J. Environ. Econ. Manag.* **98**, 102267 (2019).

10.R Zahnow, N Smith, Locality-based social media: The impact of content consumption and creation on perceived neighborhood crime, safety, and offline crime prevention. *J. Community Psychol*. **52,** 895–909 (2024).

11.F Jawadi, et al., Does higher unemployment lead to greater criminality? Revisiting the debate over the business cycle. *J. Econ. Behav. Organ*. **182**, 448–471 (2021).

12.SS Ramdayani, B Kharisma, K Wibowo, Local Government Spending on Social Protection, Security Order, and Crime. *J. Economia* **15**, 187–198 (2019).

13.S Vogl, Impacts of economic development and stability on crime: Country-level panel data analysis. *Econ. Syst*. **48**, 100283 (2024).
